# Molecular and Nano-Structural Optimization of Nanoparticulate Mn^2+^-Hexarhenium Cluster Complexes for Optimal Balance of High T_1_- and T_2_-Weighted Contrast Ability with Low Hemoagglutination and Cytotoxicity

**DOI:** 10.3390/pharmaceutics14071508

**Published:** 2022-07-20

**Authors:** Bulat Salavatovich Akhmadeev, Irek R. Nizameev, Kirill V. Kholin, Alexandra D. Voloshina, Tatyana P. Gerasimova, Aidar T. Gubaidullin, Marsil K. Kadirov, Ildus E. Ismaev, Konstantin A. Brylev, Rustem R. Zairov, Asiya R. Mustafina

**Affiliations:** 1 A.E. Arbuzov Institute of Organic and Physical Chemistry, Kazan Scientific Center, Russian Academy of Sciences, 8 Arbuzov Str., 420088 Kazan, Russia; irek.rash@gmail.com (I.R.N.); kholin06@mail.ru (K.V.K.); sobaka-1968@mail.ru (A.D.V.); tatyanagr@gmail.com (T.P.G.); aidar@iopc.ru (A.T.G.); kamaka59@gmail.com (M.K.K.); rustem02@yandex.ru (R.R.Z.); asiyamust@mail.ru (A.R.M.); 2 Department of Electronic Instrumentation and Quality Management, A.N. Tupolev Kazan Research Technological University, 420015 Kazan, Russia; iismaev@mail.ru; 3 Nikolaev Institute of Inorganic Chemistry, Siberian Branch of the Russian Academy of Sciences, 3 Acad. Lavrentiev Ave., 630090 Novosibirsk, Russia; kbrylev@gmail.com

**Keywords:** nanoparticles, manganese based contrast agents, MRI, hexarhenium clusters

## Abstract

The present work introduces rational design of nanoparticulate Mn(II)-based contrast agents through both variation of the μ_3_ (inner) ligands within a series of hexarhenium cluster complexes [{Re_6_(μ_3_-Q)_8_}(CN)_6_]^4^^−^ (Re_6_Q_8_, Q = S^2−^, Se^2−^ or Te^2−^) and interfacial decoration of the nanoparticles (NPs) K_4−2x_Mn_x_Re_6_Q_8_ (*x* = 1.3 − 1.8) by a series of pluronics (F-68, P-123, F-127). The results highlight an impact of the ligand and pluronic for the optimal colloid behavior of the NPs allowing high colloid stability in ambient conditions and efficient phase separation under the centrifugation. It has been revealed that the K_4−2x_Mn_x_Re_6_Se_8_ NPs and those decorated by F-127 are optimal from the viewpoint of magnetic relaxivities r_1_ and r_2_ (8.9 and 10.9 mM^−1^s^−1^, respectively, at 0.47 T) and low hemoagglutination activity. The insignificant leaching of Mn^2+^ ions from the NPs correlates with their insignificant effect on the cell viability of both M-HeLa and Chang Liver cell lines. The T_1_- and T_2_-weighted contrast ability of F-127–K_4−2x_Mn_x_Re_6_Q_8_ NPs was demonstrated through the measurements of phantoms at whole body 1.5 T scanner.

## 1. Introduction

Paramagnetic enhancement of transverse and longitudinal magnetic relaxation rates of water protons in aqueous solutions of ions, or complexes of paramagnetic transition metals, provides contrast-enhancement of MRI. Gd(III)-based contrast agents (CAs) are the best from the viewpoint of the accelerating of the relaxation processes, however, their side effects [1] prompt rapidly growing interest to more biogenic Mn(II)-based CAs.

Mn(II) chelates with five unpaired electrons, long electronic relaxation times and quite fast exchange rates of the coordinated water molecules provide suitable alternate to Gd(III)-based CAs, although lability of the Mn(II) chelates facilitates a release of manganese ions in bio-environment, which can also result in some neurological side effects [2,3]. Low blood circulation half-life of the manganese chelates is another issue for improvement [4]. The aforesaid problems can be solved through both diversification of an inner-sphere environment of Mn^2+^ ions [5,6,7] and nanoparticulate route in the rational design of Mn(II)-based CAs [8,9,10,11,12,13,14,15,16,17,18,19,20,21]. The route is represented in literature, but not limited by the inclusion of Mn(II) chelates into polymeric nanobeads [8,9,10,11], construction of manganese-containing metal-organic frameworks (MOFs) [12,13,14,15,16,17] and synthesis of MnO-based nanoparticles [18,19,20,21]. However, the partial leaching of Mn^2+^ ions due to endosomal/lysosomal pathway of MnO-based NPs is a reason for higher cytotoxicity of the NPs [22]. It is also worth noting that the release of Mn^2+^ ions from the MnO_2_-based nanoarchitectures in the presence of the intracellular reductants (H_2_O_2_ and glutathione) at acidic conditions is the prerequisite for the high T_1_- and T_2_-weighted contrast ability of the nanoarchitectures under their intracellular localization [23,24]. The key parameters responsible for high contrast effect of Mn^2+^-based CAs include hydration number, rotational dynamics and surface-to-volume ratio of Mn^2+^ ions, residence time of the inner-sphere water molecules [9]. The parameters can be widely diversified through modification of both nanoparticulate and colloidal parameters of NPs, which is the great advantage of the nanoparticulate CAs.

The successful use of Mn(II) complexes with inorganic ligands mainly represented but not limited by [Fe(CN)_6_]^4−^ as the building blocks of the nanoparticulate CAs has been already documented [25]. The hexarhenium chalcocyanide cluster complexes [{Re_6_(μ_3_-Q)_8_}(CN)_6_]^4−^ (Re_6_Q_8_, Q = S^2−^, Se^2−^ or Te^2−^), which are topological analogues of the hexacyanoferrate ion [Fe(CN)_6_]^4−^, represent another type of inorganic ligands able to form Mn(II)-based MOFs [26,27,28,29,30], although neither synthetic strategy for their conversion into hydrophilic NPs nor evaluation of their magnetic relaxivity has yet been reported.

It is worth noting that both incontrollable aggregation and low cell internalization of inorganic or MOF-based NPs are the issues for improvement, which can be carried out through the surface decoration of such NPs. Polyelectrolytes, oligopeptides, proteins, phospholipids are well-known efficient hydrophilic agents for composite NPs [31,32]. Moreover, high hydrophilicity of polyethylene oxide (PEO) blocks makes the so-called PEGylation of NPs promising surface decoration route, since it allows to gain in greater blood circulation time [33]. High hydrophilicity of some representatives of pluronics or triblock copolymers constituted from PEO and propylene oxide (PPO) blocks (exemplified by F-127) is also widely applied in colloid stabilization of composite NPs [34,35,36].

It is well-known that high hydrophilicity of the NPs can prevent their phase separation. This restricts key manipulations with the colloids, such as washing of the NPs from residual amounts of Mn^2+^ ions, control of their leaching from the NPs and determination of Mn-content in the NPs, which is required for accurate measuring of r_1_ and r_2_ values. Triblock copolymers exhibit unique temperature-dependent aggregation behavior, which can be greatly modified through the lengths’ variation of PEO and PPO blocks [37]. This opens new opportunities in controlling the size of the NPs and their temperature-dependent behavior. The triblock copolymers have been revealed as efficient hydrophilic agents in the synthesis of the nanoparticulate Gd^3+^-based MOFs with high contrasting ability [38]. However, an impact of their structure and temperature-induced aggregation on controlling of size, aggregation and magnetic relaxation behavior of the MOF-based CAs is not well recognized.

Thus, the present work introduces the complex formation of Mn^2+^ ions with [{Re_6_(μ_3_-Q)_8_}(CN)_6_]^4−^ as the synthetic approach to generate K_4−2x_Mn_x_Re_6_Q_8_-based hydrophilic NPs. The advantage of the using F-127 as the hydrophilic agent vs. another triblock copolymers, namely F-68 and P-123, in the hydrophilic coating of the NPs is demonstrated. The specific role of F-127 on the relaxivity (r_1_ and r_2_) values of the NPs is also shown through the magnetic relaxation measurements at 0.47 T and 1.5 T. The T_1_- and T_2_-weighted contrast ability of the colloids is compared with that of the commercial CA Omniscan by means of whole-body scanner at 1.5 T. The measurements reveal low hemoagglutination activity of K_4−2x_Mn_x_Re_6_Q_8_-based hydrophilic NPs, which is the prerequisite for longer blood circulation half-life of the NPs. This, in turn, argues for further in vivo applicability of K_4−2x_Mn_x_Re_6_Q_8_-based hydrophilic NPs.

## 2. Experimental Section

### 2.1. Materials

MnCl_2_, phosphate buffer solution, triblock copolymer (F-127, P-123, F-68), and Xylenol orange were purchased from Sigma Aldrich.

Potassium salts of the hexarhenium cluster complexes K_4_[{Re_6_Q_8_}(CN)_6_] (Q = S^2−^, Se^2−^ or Te^2−^) were synthesized in accordance with previous works [27].

### 2.2. Methods

The detailed description of the common methods (dynamic light scattering (DLS), powder X-ray diffraction (PXRD), inductively coupled plasma optical emission spectrometry (ICP-OES), transmission electron microscopy (TEM), UV-Vis, IR and electronic spin resonance spectroscopy) are in the Supplementary Materials.

### 2.3. Relaxometry

The proton relaxation times T_1_ and T_2_ were measured using pulsed NMR-relaxometer Minispec MQ20 from Bruker with operational frequency of 19.65 MHz (0.47 T) by applying the standard radio frequency pulse sequences: inversion-recovery method (spin-lattice relaxation time T_1_), and Carr-Purcell sequence, modified by Meiboom-Gill (spin-spin relaxation time T_2_) with the measuring accuracy error smaller than 3%. The temperature was maintained with the Thermo/Haake DC10 circulator.

The contrasting ability of the as-prepared Mn-containing colloids was demonstrated through the T_1_- and T_2_-weighted images obtained by means of whole body 1.5 T scanner (Excel Art Vantage Atlas X, Toshiba, Otawara, Japan) equipped with 65-cm horizontal bore size corresponding to a proton resonance frequency of 63.58 MHz. The detailed description of the measuring procedure is in the Supplemetary Materials.

### 2.4. Synthesis of K_4−2x_Mn_x_Re_6_Q_8_

Aqueous solutions of [{Re_6_Q_8_}(CN)_6_]^4−^ (Q = S^2−^, Se^2−^ and Te^2−^) (2 mM) were prepared and acidified to pH~5 by adding of 20 µL of HCl (0.1 M) per 1 mL of the solution. The aqueous solution (200 μL) of MnCl_2_ (10 mM) was added dropwise by syringe pump (100 μL/min) to the mixture of the solutions of [{Re_6_Q_8_}(CN)_6_]^4−^ (1 mL, 2 mM) and of pluronic (0.5 mL, 1 g∙L^−1^).

The as-synthesized Mn_x_Re_6_Q_8_ NPs were precipitated in a centrifuge at 14,500 rpm for 40 min at a temperature of 310–318 K. The separated NPs were redispersed in 3.4 mL of the pluronic’s solutions (1 g∙L^–1^) through the ultrasonication for 10 min at 288 K.

### 2.5. Determination of Re:Mn Ratio

The spectrophotometric determination of Mn^2+^ content in the supernatants is based on its complexation with xylenol orange (XO) [39,40] at pH = 6.8, which leads to increase of intensity of the absorption band at 580 nm. The standard solution of XO (1 mM) was prepared (see ESI). Three solutions were prepared: 1—reference solution of XO (2.9 mL of phosphate buffer + 0.1 mL of XO); 2—measurement solution (2.8 mL of phosphate buffer + 0.1 mL of XO + 0.1 mL of the supernatant, obtained after precipitation of Mn_x_Re_6_Q_8_); 3—reference solution of the Mn-XO complex (2.8 mL of phosphate buffer + 0.1 mL of XO + 0.1 mL of MnCl_2_ (0.588 mM), which simulate 100% manganese in the system). UV-vis spectra were recorded for all solution (Figure S1).

The residual amount of Mn^2+^ (%) in the supernatant was determined by the formula:

(1) $$C\left(\mathrm{st}\right)=\frac{\left(A_{2}^{580}-A_{XO}^{580}\right)}{\left(A_{3}^{580}-A_{XO}^{580}\right)}*100\%$$

The concentration of manganese in the particles was determined as C_max_ * (100—C(st)), where C_max_ is concentration of Mn^2+^ in initial solution before centrifugation.

UV-vis spectra of [{Re_6_Q_8_}(CN)_6_]^4^^−^ (Q = S^2−^, Se^2−^ or Te^2−^) at different concentration were obtained. The concentrations of [{Re_6_Q_8_}(CN)_6_]^4–^ in supernatant were determined by linear dependences (Figure S2).

### 2.6. Hemagglutination Assay

Human erythrocytes were washed twice with physiological saline (NaCl 0.9%) and centrifuged at 2500× *g* for 10 min at 4 °C. After each cycle, the supernatant was carefully removed. Then the red blood cells were resuspended in physiological saline to a concentration of 2%. Hemagglutination activity was analyzed in a 96-well U-plate. Double dilution series of the aqueous colloids of K_4−2x_Mn_x_Re_6_S_8_ and K_4−2x_Mn_x_Re_6_Se_8_ were prepared. 100 µL of the colloids was mixed with 100 µL of 2% red blood cell solution and put into a well. Each concentration point was carried out simultaneously in two wells in parallel. After 1 h of incubation at 37 °C, hemagglutination was observed with unaided eye [41] and checked using Nikon Eclipse Ci-S fluorescence microscope (Nikon, Nasu Nasu-Gun, Japan) with Ph1 condenser ring in phase contrast mode. A suspension of red blood cells in 0.9% saline and a mixture of type A(II) and C(IV) erythrocytes were used as negative and positive agglutination control, respectively.

## 3. Results and Discussion

### 3.1. Synthesis and Characterization of K_4−2x_Mn_x_Re_6_Q_8_

Literature data indicate that coordination of Mn^2+^ ions with [{Re_6_Q_8_}(CN)_6_]^4^^−^ (Re_6_Q_8_, Q = S^2−^, Se^2−^ or Te^2−^) generates great diversity of MOF-like structures, where the framework derives from Re–C–N–Mn bridges, while the binding still remains the opportunity of the Mn^2+^ ions to coordination of water, solvent molecules or chelating ligands [27]. According to these reports the mixing of manganese salts with potassium salts of [{Re_6_Q_8_}(CN)_6_]^4−^ in aqueous solutions triggers a formation of MOF-like supramolecular structures, which can be applied in generation of MOF-based NPs under the conditions of controlling the size of the formed NPs. The facile route to control the size was previously demonstrated for the MOF-based nanoparticles constructed from the [{Re_6_Q_8_}(CN)_6_]^4−^ and Gd^3+^ ions with the use of F-127 as hydrophilic agent [42]. However, problems with a phase separation of ultra-small NPs coated by hydrophilic shell indicate a necessity for a balance between high colloid stability of hydrophilic NPs and their ability to easy phase separation. The use of triblock copolymers as hydrophilic agents provides an ability to control colloidal behavior of the Mn(II)-based NPs by managing of their hydrophilic shell.

**Figure 1:**
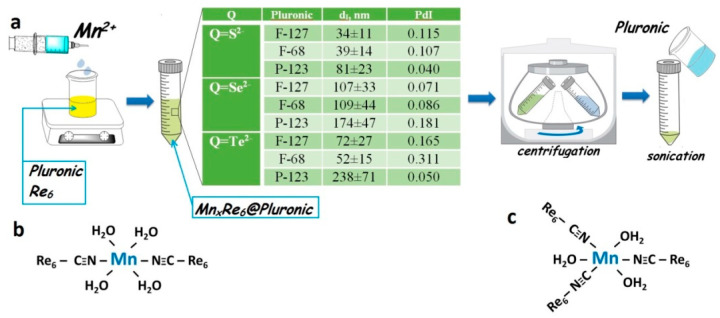
(**a**) Schematic presentation of the synthetic strategy applied to obtain K_4−2x_Mn_x_Re_6_Q_8_-based NPs, the most probable structural motifs of binding of the Mn^2+^ ions with the cyanide groups of the clusters are schematically shown (**b**,**c**).

The structure diversity of triblock copolymers derived from the lengths’ variation of PEO and PPO blocks allows to modify their self-aggregation in aqueous solutions. The Mn(II)-based NPs were obtained for [{Re_6_Q_8_}(CN)_6_]^4−^)Q = S^2−^, Se^2−^ or Te^2−^) in the solutions of (PEO)_100_-(PPO)_64_-(PEO)_100_ (F-127), (PEO)_75_(PPO)_30_(PEO)_75_ (F-68), (PEO)_20_(PPO)_70_(PEO)_20_ (P-123), which concentrations were adjusted at 1 g∙L^−1^. The synthetic stages are schematically demonstrated in Figure 1a along with the most probable structural motifs of the binding of the Mn^2+^ ions with the cluster units (Figure 1b,c). The comparative analysis of the synthetic data at various structure and concentration of the components reveals the conditions optimal for formation and separation of the NPs, which were further characterized by elemental analysis, TEM, PXRD, DLS techniques (Table 1).

The stoichiometry of the heterometallic NPs was determined through both spectrophotometric XO-assisted analysis of supernatants and ICP-OES analysis of the separated colloids (Table S1). The Mn:Re_6_ ratios determined by the methods are very close, indicating that the values are about 1.3, 1.8 and 1.8 for Re_6_Q_8_ at Q = S^2−^, Se^2−^ and Te^2−^, respectively (Table 1). This confirms heterometallic nature of the NPs, which herein and further will be designated as K_4−2x_Mn_x_Re_6_Q_8_.

It is worth noting that F-127 and F-68 provide enough stabilization of the NPs, while the aggregation is significantly greater for P-123 (Table 1). The concentration of the triblock copolymers at the level of 1 g∙L^−1^ corresponds to 0.08 mM of F-127, 0.12 mM of F-68 and 0.17 mM of P-123. Thus, only P-123 forms the micellar aggregates in the solutions at 295 K [37], while the CMC (critical concentration of micellization) values of F-127 and F-68 in these conditions are above the applied concentrations [43,44]. Thus, the triblock copolymer molecules should be “free” for interfacial stabilizing of the NPs, while the self-aggregation of the triblock copolymers decreases their capacity to hydrophilize the NPs.

**Figure 2:**
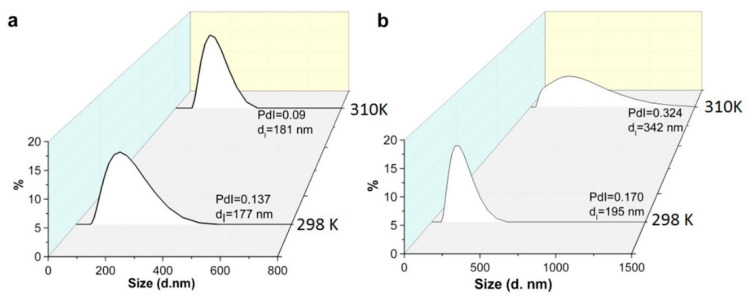
Size distribution by intensity for K_4−2x_Mn_x_Re_6_Q_8_, stabilized by F-127 {(PEO)_100_-(PPO)_64_-(PEO)_100_} (**a**) and P-123 {(PEO)_20_(PPO)_70_(PEO)_20_} (**b**) at 298 K and 310 K.

The aforesaid allows to choose F-127 and F-68 as more convenient hydrophilic agents than P-123, although the centrifugation-induced phase separation is very poor at ambient temperatures. It is worth noting the temperature-dependent micelle formation is the key specificity of the aggregation behavior of the triblock copolymers [37,44]. In particular, the CMC of F-127 decreases to 0.0028 mM at 303 K [45], while the temperature of micellization is above 323 K for F-68 (1 g∙L^−1^) [44]. This indicates that the temperature rise under the centrifugation conditions can facilitate the phase separation of the NPs. Indeed, the temperature level at 303 K is enough to reach complete phase separation of the NPs from the F-127-based solutions under the centrifugation (for more details see Section 2), while the insufficient phase separation of the NPs is observed from the solutions of F-68 at the same temperature. Thus, the heating to 303 K decreases an extent of the “free” triblock copolymers due to their aggregation into micelles. This can restrict a participation of the triblock copolymers in hydrophilic coating of the NPs, thus, facilitating their phase separation under the centrifugation. In order to recognize a specific temperature-dependent effect of F-127 on the aggregation behavior of the NPs the latter was monitored at various temperatures in the solutions of P-123, F-68, F-127 (1 g∙L^−1^). The DLS data (Figure 2) reveal the detectable aggregation of the NPs in the solutions under their heating to 303 K for F-127 and P-123, which is enough for the centrifugation-induced phase separation of K_4−2x_Mn_x_Re_6_Q_8_. The lack of detectable aggregation observed for F-68 correlates with the poor phase separation of the NPs in the same conditions. This confirms the aforesaid assumption that the ability of the NPs to phase separation can be controlled by the aggregation behavior of the triblock copolymers. It is worth noting that the developed synthetic procedure is reproducible (has been tested for six times at least), facile and not time consuming, in particular, the synthesis takes no more than 1.5 h.

**Figure 3:**
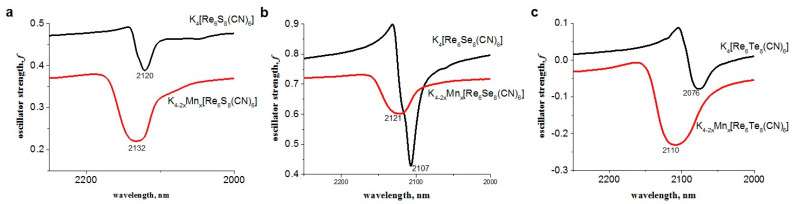
IR-spectra of K_4_[{Re_6_Q_8_ (CN)_6_] and K_4−2x_Mn_x_Re_6_Q_8_: Q = S^2−^ (**a**); Q = Se^2−^ (**b**); Q = Te^2−^ (**c**).

The separated colloids were dried and characterized by IR spectroscopy with the focus on the bands arisen from the apical cyanide ligands of the cluster complexes, since their binding with Mn^2+^ ions should be followed by the shifting of maxima of the bands to higher energies as it was exemplified by [27]. Indeed, the shifting was revealed from the comparison the bands attributed to CN-groups in the K_4−2x_Mn_x_Re_6_Q_8_ NPs with those of the corresponding K_4_[{Re_6_Q_8_}(CN)_6_] salts (Figure 3 and Figure S3). This confirms that Mn^2+^ ions are coordinated with the apical cyanides of the Re_6_Q_8_ cluster units. The residual amounts of F-127 molecules are also revealed from the IR spectra (Figure S3).

**Figure 4:**
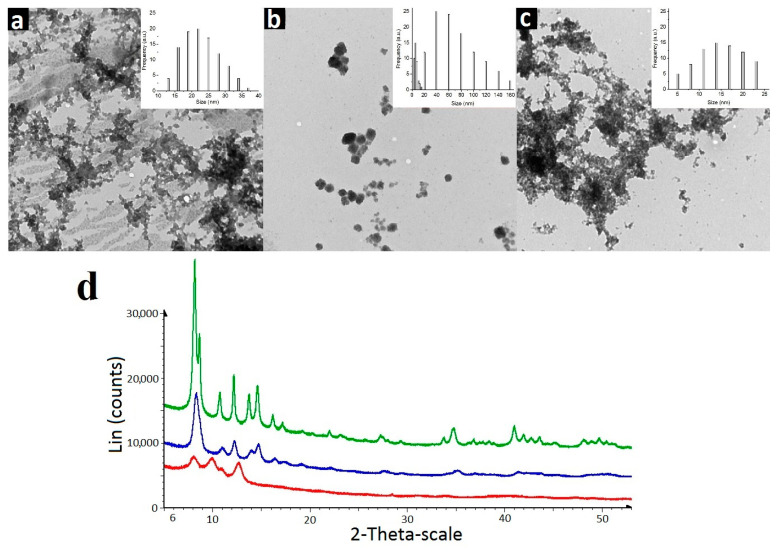
TEM images of dried colloids K_4−2x_Mn_x_Re_6_Q_8_, where Q = S^2−^ (**a**), Se^2−^ (**b**), Te^2−^ (**c**), the insets represent the size distribution. (**d**)-experimental diffraction patterns of K_4−2x_Mn_x_Re_6_Q_8_ (Q = Te^2−^ (red curve), Q = S^2−^ (blue curve) and Q = Se^2−^ (green curve).

PXRD analysis of dried colloids reveals different extent of crystallinity of the colloids (Figure 4). The similarity in the PXRD patterns of K_4−2x_Mn_x_Re_6_S_8_ and K_4−2x_Mn_x_Re_6_Se_8_ colloids indicates the isostructural nature of their crystalline forms. The higher crystallinity of K_4−2x_Mn_x_Re_6_Se_8_ colloids is followed by the bigger size of their nanocrystallites being within 20–42 nm (Figure S4, Table S2 in ESI), which agrees well with the size distribution from TEM analysis (Figure 4). The size-values evaluated for K_4−2x_Mn_x_Re_6_S_8_ and K_4−2x_Mn_x_Re_6_Te_8_ from PXRD data are 11–16 and 7–12 nm, respectively (Figures S5 and S6, Tables S2 and S3), which also close to the size-values revealed by the TEM analysis (Figure 4). The PXRD pattern of K_4−2x_Mn_x_Re_6_Te_8_ crystallites indicates that their diffraction pattern is closer to that of nanostructured systems (Figure 4d) due to the low crystallizing ability of K_4−2x_Mn_x_Re_6_Te_8_ and very small crystallite sizes. However, a comparison of the positions of the observed interference peaks allows to state that the crystals of K_4−2x_Mn_x_Re_6_Te_8_ colloids are not isostructural to the sulfide- and selenide-counterparts. It is worth noting that the peculiar crystal packing was also revealed for the gadolinium complexes of [{Re_6_Te_8_(CN)_6_]^4−^ cluster [42].

It is worth noting that the as-separated colloids being further redispersed in “pure” water suffer from the instability manifested by the precipitation within one hour, since the residual amounts of F-127 cannot provide the colloidal stability of K_4−2x_Mn_x_Re_6_Q_8_. Thus, the separated K_4−2x_Mn_x_Re_6_Q_8_-based NPs should be further dispersed in aqueous solutions of the triblock copolymers (F-127, F-68 or P-123) for the high colloid stability at both ambient (298 K) and physiological (310–318 K) temperature. The aqueous colloids of K_4−2x_Mn_x_Re_6_Q_8_ generate sextet in the ESR spectra (Figure S7) with the linewidths ΔH = 21.5 G, hyperfine coupling constant a_Mn_ = 95 G and g = 2.002, which is peculiar for Mn^2+^ ions, while the linewidth values argue for the interionic interactions derived from the package of K_4−2x_Mn_x_Re_6_Q_8_ complexes into the NPs. The ESR spectral features of the NPs indicate their d^5^ electronic structure confirming its prospect for paramagnetic enhancement of the magnetic relaxation of water protons in their aqueous dispersions.

### 3.2. Magnetic Relaxivity of K_4−2x_Mn_x_Re_6_Q_8_

**Figure 5:**
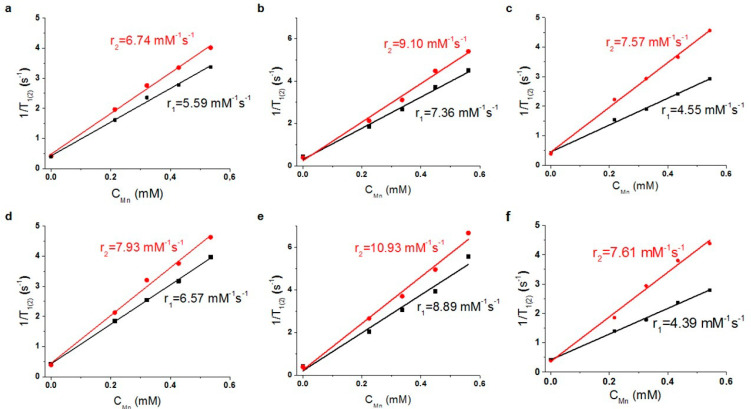
1/T_1(2)_ vs. Mn^2+^ concentration, measured for F-127-K_4−2x_Mn_x_Re_6_Q_8_ at 298 K (**a**–**c**) and 310 K (**d**–**f**): Q = S^2−^ (**a**,**d**), Q = Se^2−^ (**b**,**e**), Q = Te^2−^ (**c**,**f**).

The longitudinal and transverse relaxation rates of water protons are enhanced in the aqueous colloids of K_4−2x_Mn_x_Re_6_Q_8_, and the rates exhibit linear increase with the concentration growth of K_4−2x_Mn_x_Re_6_Q_8_ (Figure 5 and Figure S3). This confirms low aggregation of the as-prepared NPs and allows to calculate the r_1_ and r_2_ values. It is worth noting that the linearity is observed for the rates measured at both 298 and 310 K, and the r_1(2)_ values measured at 310 K are greater than those measured at 298 K. The observed temperature-induced relaxation enhancements refer to the condition T_1_ < τ_M_, where the relaxivity enhanced by the factor τ_R_ is retarded by the slow water exchange process [46]. This agrees well with the nanoparticulate form of K_4−2x_Mn_x_Re_6_Q_8_ complexes, which is the reason for a relaxivity enhancement due to long molecular reorientational time τ_R_. However, the supramolecular packing of K_4−2x_Mn_x_Re_6_Q_8_ complexes into the NPs can restrict the accessibility of Mn^2+^-centers to efficient exchange of the inner-sphere water molecules with the bulk. It is well-known that the water exchange is most efficient for the interfacial complexes, thus, surface-to-volume ratio should be of great impact on the relaxivity. Also, it is well-known that ultra-small size below 10 nm provides more efficient paramagnetic enhancement of water protons than that derived from the greater sized NPs [47]. However, the r_1_ and r_2_ values are the greatest for K_4−2x_Mn_x_Re_6_Se_8_ vs. those for K_4−2x_Mn_x_Re_6_S_8_ and K_4−2x_Mn_x_Re_6_Te_8_, which disagrees with the size values of the corresponding NPs evaluated from both PXRD and TEM measurements. In turn, the average size values measured for the NPs in the F-127 based solutions by the DLS technique indicate their aggregation (Table 1). Thus, the aggregation event can significantly level the deviations arisen from the different initial size of the NPs.

**Figure 6:**
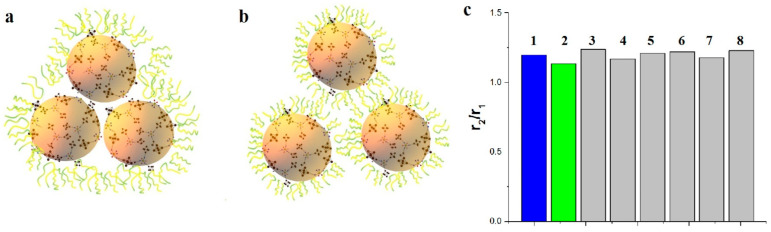
Schematic representation of the different aggregation modes, that are formed during stabilization K_4−2x_Mn_x_Re_6_Se_8_ by P-123 (**a**) and F-127 (**b**). (**c**)—r_2_/r_1_ values, measured in water solution (1), in phosphate buffer (10 mM) solution with BSA (1 g·L^−1^) (2) and in phosphate buffer (10 mM) solution under various time of storage: 1 day (3), 2 days (4), 5 days (5), 6 days (6), 7 days (7) 10 days (8).

The r_1_ and r_2_ values of K_4−2x_Mn_x_Re_6_Se_8_ dispersed in the solutions of different triblock copolymers depend on their nature, being the smallest for P-123, while growing in the following series P-123 < F-68 < F-127 (Table 1). The oxidation state of the interfacial Mn^2+^ ions can change to Mn^3+^ in the ambient conditions without deoxygenation, which is commonly observed for MnO-based NPs [47], but the oxidation state cannot be the reason for the tendency. It is worth assuming that the tendency derives from the different participation of the triblock copolymer molecules in hydrophilic coating of K_4−2x_Mn_x_Re_6_Q_8_, although the average size values measured at 298 K reveal poor dependence on the triblock copolymer’s nature. The lowest r_1_ and r_2_ values observed in the solutions of P-123 argues for the lowest accessibility of the Mn^2+^ ions to hydration, while the highest r_1_ and r_2_ values in the F-127-based solution correlate with the most efficient hydrophilic coating of K_4−2x_Mn_x_Re_6_Q_8_ by F-127. It is also worth noting that the deviation between the r_1_ and r_2_ values arisen from the size and composition of the cores is on the same level of magnitude with the deviations between the values measured for different triblock copolymers. The aforesaid allows to hypothesize at least two types of the aggregation modes of K_4−2x_Mn_x_Re_6_Q_8_ in the solutions of F-127 and P-123 as it is schematically illustrated in the cartoon image (Figure 6). The first type represented by the agglomerated NPs coated by the total hydrophilic shell (Figure 6a) in the greater extent restricts an accessibility of the Mn^2+^ ions to hydration than the second one manifested by a self-assembly of the hydrophilic NPs (Figure 6b). Actually, both modes of the aggregation can be characterized by the similar sizes, but diverse r_1_ and r_2_ values (Table 1). The predominance of the second aggregation mode correlates with the ability of the triblock copolymer to form a hydrophilic coating of the NPs, being the most for F-127.

The r_2_/r_1_ ratios are greatly affected by the hydration number (q) of Mn(II) complexes, since longitudinal and transverse relaxation rates are differently contributed by the scalar mechanism [14]. Thus, r_2_/r_1_ ratio is the greatest (4.8) for Mn(II) aqua ions, while comes to minimum (1.2) when q = 0 [48]. The ratios for K_4−2x_Mn_x_Re_6_S_8_ and K_4−2x_Mn_x_Re_6_Se_8_ lie within 1.21–1.24 in the solutions of the triblock copolymers independently on their nature, while the greater ratios are revealed for K_4−2x_Mn_x_Re_6_Te_8_ (Table 1). The tendency agrees well with the PXRD data (Figure 4) revealing isostructural features of K_4−2x_Mn_x_Re_6_S_8_ and K_4−2x_Mn_x_Re_6_Se_8_, while the ratios 1.66–1.73 of K_4−2x_Mn_x_Re_6_Te_8_ correlate with its structural specificity. Thus, the structural features of K_4−2x_Mn_x_Re_6_Q_8_ significantly affect the r_2_/r_1_ ratio, while their effect on the r_1_ and r_2_ values is distorted by the influence of the aggregation behavior of K_4−2x_Mn_x_Re_6_Q_8_ in the aqueous solutions.

**Figure 7:**
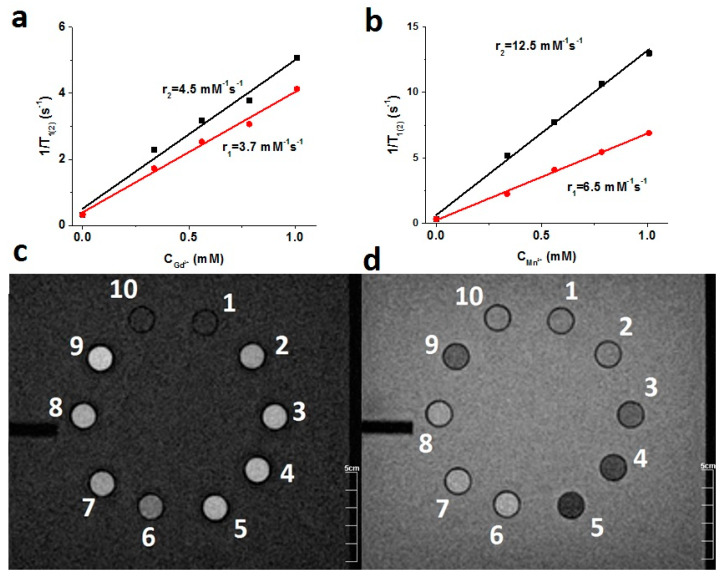
1/T_1(2)_ values vs. Gd(III) concentration (**a**) and Mn(II) concentration (**b**), measured at 1.5 T and 296 K for Omniscan (**a**) and F-127-K_4−2x_Mn_x_Re_6_Se_8_ (**b**). (**c**,**d**) T_1_- and T_2_-weighted images of vials with F-127-K_4−2x_Mn_x_Re_6_Se_8_ (2–5) and Omniskan (6–9) at different concentration of Mn^2+^ and Gd^3+^: 1,10—water; 2—0.336 mM Mn^2+^; 3—0.560 mM Mn^2+^; 4—0.784 mM Mn^2+^; 5—1.008 mM Mn^2+^; 6—0.336 mM Gd^3+^; 7—0.560 mM Gd^3+^; 8—0.560 mM Gd^3+^; 9—1.008 mM Gd^3+^.

The r_1_ values of F-127–K_4−2x_Mn_x_Re_6_Se_8_ colloids are greater than those reported for the Gd-containing commercial CAs, while the r_2_/r_1_ values of the colloids are below 2 (Table 1) similar with the Gd-containing CAs [47]. Since the magnetic field strengths also influence both r_1(2)_ and r_2_/r_1_ values of nanoparticulate CAs [47], the literature values measured at 0.5 T are required for the correct comparison with the r_2_/r_1_ values collected in Table 1. The comparison indicates that the represented r_2_/r_1_ values are the least among those reported for the Mn-containing nanoparticulate CAs at 0.5 T [49,50]. The measurements at 1.5 T by means of the whole-body scanner reveal the following relaxivity values r_1(2)_ = 6.5(12.8) mM^−1^s^−1^ and r_2_/r_1_ = 1.96 for F-127–K_4−2x_Mn_x_Re_6_Se_8_. The values measured for Omniskan by the same equipment (r_1(2)_ = 3.7(4.5) mM^−1^s^−1^) agree well with the literature values [51] (Figure 7). The comparison of the latter values with those of F-127–K_4−2x_Mn_x_Re_6_Se_8_ colloids reveals their advantage vs. the commercial Gd-containing CAs. The r_1(2)_ values of F-127–K_4−2x_Mn_x_Re_6_Se_8_ are close to the best reported in literature values of Mn-containing CAs (r_1(2)_ = 8.4(16.8) mM^−1^s^−1^ and r_2_/r_1_ = 2.0) measured at 1.5 T [47], however, the r_2_/r_1_ values of the Mn-containing nanoparticulate CAs measured at magnetic field strength greater than 1.5 T are far above 2.0 [10,52,53]. The level of r_2_/r_1_ about 2 is convenient for the low interference of T_1_-weighted relaxivity by the T_2_-weighted one [47].

### 3.3. Leaching, Cytotoxicity, Hemagglutination Assay and Imaging Capacity of K_4−2x_Mn_x_Re_6_Se_8_

The leaching of Mn^2+^ ions from K_4−2x_Mn_x_Re_6_Se_8_ buffered solutions of BSA modeling blood plasma can be easily monitored through r_2_/r_1_ ratio, since the ratio for aqua Mn^2+^ ions is ~4.8 [48], which is substantially higher than that of the NPs in aqueous solutions. Thus, the remaining unchanged of the r_2_/r_1_ ratio of K_4−2x_Mn_x_Re_6_Se_8_ in the buffered solutions within ten days (Figure 6c) indicates the insignificant (below 5% of the total concentration of Mn) leaching of Mn^2+^ ions from the NPs. The invariance of the DLS measurements of F-127-K_4−2x_Mn_x_Re_6_Se_8_ colloids performed within one month (Figure S8) confirms their chemical and colloidal stability.

The insignificant leaching well correlates with no effect of K_4−2x_Mn_x_Re_6_S_8_ and K_4−2x_Mn_x_Re_6_Se_8_ on the cell viability of both M-HeLa and Chang Liver cell lines (Table S5), where the cell viability values does not reach 50% even at 90 μM of the NPs.

**Figure 8:**
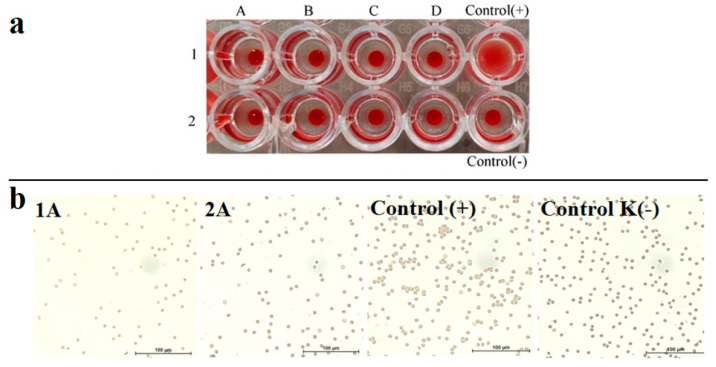
(**a**)—Hemagglutination activity of F-127–K_4−2x_Mn_x_Re_6_S_8_ (1) and F-127–K_4−2x_Mn_x_Re_6_Se_8_ (2) at various manganese concentrations: 10 μM (A), 20 μM (B), 50 μM (C), 100 μM (D). (**b**)—effect of K_4−2x_Mn_x_Re_6_S_8_ (1A) and K_4−2x_Mn_x_Re_6_Se_8_ (2A) on agglutination of erythrocytes was observed by fluorescent microscopy in phase contrast mode at the highest colloid concentration (0.1 mM). Positive agglutination control derives from mixture of type A(II) and C(IV) erythrocytes—positive agglutination control, while negative control is provided by nontreated cells.

Intravenous applicability of CAs raises a question about their hemocompatibility [54]. The hemagglutination assay has been performed to reveal hemocompatibility of K_4−2x_Mn_x_Re_6_S_8_ and K_4−2x_Mn_x_Re_6_Se_8_ at the concentrations varying from 10 to 100 μM. It can be seen from Figure 8a that a round red button is present at the bottom of the wells filled by erythrocytes in physiological saline and mixed with the colloids, which corresponds to a negative reaction, while a carpetlike structure is peculiar for agglutinating erythrocytes (positive control). This indicates the lack of hemagglutination even at the greatest concentrations of K_4−2x_Mn_x_Re_6_S_8_ and K_4−2x_Mn_x_Re_6_Se_8_.

The microscopic images of the erythrocytes mixed with the aqueous colloids of K_4−2x_Mn_x_Re_6_S_8_ and K_4−2x_Mn_x_Re_6_Se_8_ at the highest colloid concentration demonstrated in Figure 8b also reveal very poor agglutination if any. It is worth explaining the low hemagglutination activity of the F-127–K_4−2x_Mn_x_Re_6_Q_8_ colloids by the hydrophilic PEO chains constituting their exterior coating. This, in turn, is a good prerequisite for a long blood circulation half-life time under in vivo application of the colloids.

## 4. Conclusions

Summarizing, the complex formation of Mn^2+^ ions with [{Re_6_Q_8_}(CN)_6_]^4−^ (Re_6_Q_8_, Q = S^2−^, Se^2−^ or Te^2−^) is for the first time represented as the facile route to generate heterometallic nanoparticles K_4−2x_Mn_x_Re_6_Q_8_, where *x* = 1.3–1.8. The results highlighted the temperature-dependent aggregation behavior of the triblock copolymers (F-127, F-68 and P-123) as the key factor for stabilizing of K_4−2x_Mn_x_Re_6_Q_8_ colloids in ambient conditions and their phase separation under the centrifugation. The F-127 fits well to the aforesaid requirements, thus, the treating by F-127 of K_4−2x_Mn_x_Re_6_Q_8_ colloids allows to gain in high colloid stability and facile phase separation. The variation of the ligand’s structure revealed the [{Re_6_Se_8_}(CN)_6_]^4−^ cluster complex as the optimal one for a combination of the magnetic relaxivity values (r_1_ = 8.9 and r_2_ = 10.9 mM^−1^s^−1^ at 0.47 T and 310 K) with the low levels of hemoagglutination activity and cytotoxicity of the colloids. Thus, the reported herein r_1_ and r_2_ values of F-127–K_4−2x_Mn_x_Re_6_Q_8_ colloids are not among the leaders, but their r_2_/r_1_ values are within 1.2–1.7, which is the advantage of the colloids differentiating them from the documented in literature nanoparticulate Mn-containing CA_S_. Thus, both molecular and nano-structural optimization of K_4−2x_Mn_x_Re_6_Q_8_ colloids was successful for optimal balance of high T_1_- and T_2_-weighted contrast ability with an applicability for in vivo imaging of the colloids derived from their low hemoagglutination and cytotoxicity.

## Figures and Tables

**Table 1:** Mn:Re_6_Q_8_ ratio, the magnetic relaxivity values (r_1_, r_2_ and r_2_/r_1_) of F-127(F-68 and P-123)–K_4−2x_Mn_x_Re_6_Q_8_ (Q = S^2−^, Se^2−^ or Te^2−^) colloids and their d_av_ (average size) and PDI (polydispersity indices) values evaluated by DLS data at various temperatures.

	Mn:Re_6_Q_8_ Ratio (x)	r_1_, mM^−1^s^−1^	r_2_, mM^−1^s^−1^	r_2_/r_1_	PDI	d_av_, nm (DLS)	d nm (TEM)	T, K
F-127–K_4−2x_Mn_x_Re_6_S_8_	1.3	5.59	6.74	1.21	0.268	162 ± 51	20 ± 8	298
		6.57	7.93	1.21				310
F-127–K_4−2x_Mn_x_Re_6_Se_8_	1.8	7.36	9.1	1.24	0.157	191 ± 23	50 ± 37	298
		8.89	10.93	1.23	0.09	181 ± 33		310
F-127–K_4−2x_Mn_x_Re_6_Te_8_	1.8	4.55	7.57	1.66	0.205	114 ± 48	15 ± 7	298
		4.39	7.61	1.73				310
F-68–K_4−2x_Mn_x_Re_6_Se_8_		6.49	7.90	1.22	0.110	178 ± 57		298
		7.71	9.55	1.24	0.127	180 ± 65		310
P-123–K_4−2x_Mn_x_Re_6_Se_8_		4.91	6.00	1.22	0.170	195 ± 74		298
		5.89	7.22	1.23	0.324	342 ± 110		310

## Data Availability

Reported results can be found in archived datasets of A.E. Arbuzov Institute of Organic and Physical Chemistry, Kazan Scientific Center, Russian Academy of Sciences, 8 Arbuzov Str., 420088 Kazan, Russia.

## Supplementary Materials

The following supporting information can be downloaded at: https://www.mdpi.com/article/10.3390/pharmaceutics14071508/s1, Figure S1: UV-vis spectra of Xylenole orange (black line), Xylenole orange with 1.17 mM Mn(II) (pink line), of Xylenole orange with supernatant, obtained after sedimentation K_4−2x_Mn_x_Re_6_Q_8_-based hydrophilic NPs: Q = S^2−^ (red line), Q = Se^2−^ (blue line), Te^2−^ (line); Figure S2: I/I_0_ vs concentration of [Re_6_Q_8_(CN)_6_]^4−^ for Q = S^2−^ (a), Se^2−^ (b), Te^2−^ (c). UV-vis spetra of Re_6_Q_8_(CN)_6_]^4−^ for Q = S^2−^ (d), Se^2−^ (e), Te^2−^ (f). UV-vis spetra of supernatant, obtained after centrifugation K_4−2x_Mn_x_Re_6_Q_8_-based hydrophilic NPs: Q = S^2−^ (g), Q = Se^2−^ (h), Te^2−^ (f); Table S1: XO-assisted and ICP-OES analysis of K_4−2x_Mn_x_Re_6_Q_8_-based hydrophilic NPs; Figure S3: IR spectra of K_4-2x_Mn_x_Re_6_Q_8_-based NPs and [Re_6_Q_8_(CN)_6_]^4−^; Figure S4: Powder XRD pattern of K_4−2x_Mn_x_Re_6_Q_8_, Q = Te^2−^ complex, experimental (blue), calculated (red) and their difference (grey) curves are depicted; Figure S5: Powder XRD pattern of K_4−2x_Mn_x_Re_6_Q_8_, Q = Se^2−^ complex, experimental (green), calculated (red) and their difference (grey) curves are depicted; Figure S6: Powder XRD pattern of K_4−2x_Mn_x_Re_6_Q_8_, Q = S^2−^ complex, experimental (black), calculated (red) and their difference (grey) curves are depicted; Table S2: Crystallite size, calculated from experimental diffraction patterns of K_4−2x_Mn_x_Re_6_Q_8_, Q = Se^2−^; Table S3: Crystallite size, calculated from experimental diffraction patterns of K_4−2x_Mn_x_Re_6_Q_8_, Q = S^2−^ complex; Table S4: Crystallite size, calculated from experimental diffraction patterns of K_4−2x_Mn_x_Re_6_Q_8_, Q = Te^2−^; Figure S7: ESR spectra of of F-127-K_4−2x_Mn_x_Re_6_Q_8_: Q = S^2−^ (a); Q = Se^2−^ (b); Q = Te^2−^ (c); Figure S8: Size distribution by volume of F-127-K_4−2x_Mn_x_Re_6_Q_8_ at initial time (black line) and after 7 day (red line); Table S5: Cell viability of F-127-K_4−2x_Mn_x_Re_6_Q at different manganese concentrations. References [55,56] are cited in the supplementary materials.
