# Supplementary material for: Molecular and Nano-Structural Optimization of Nanoparticulate Mn2+-Hexarhenium Cluster Complexes for Optimal Balance of High T1- and T2-Weighted Contrast Ability with Low Hemoagglutination and Cytotoxicity
Source: Pharmaceutics. 2022 Jul 20;14(7):1508. doi: 10.3390/pharmaceutics14071508 (PMC9316779; doi:10.3390/pharmaceutics14071508)
Supplement: Supplementary file 1 [file pharmaceutics-14-01508-s001.zip › pharmaceutics-1783819-supplementary.pdf]

Molecular and Nano-Structural Optimization of Nanoparticulate Mn^{2+}-Hexarhenium Cluster Complexes for Optimal Balance of High T_1- and T_2-Weighted Contrast Ability with Low Hemoagglutination and Cytotoxicity

Bulat Salavatovich Akhmadeev [1,*], Irek R. Nizameev [1], Kirill V. Kholin [1], Alexandra D. Voloshina [1], Tatyana P. Gerasimova [1], Aidar T. Gubaidullin [1], Marsil K. Kadirov [1], Ildus E. Ismaev [2], Konstantin A. Brylev [3], Rustem R. Zairov [1] and Asiya R. Mustafina [1]

[1] A.E. Arbuzov Institute of Organic and Physical Chemistry, Kazan Scientific Center, Russian Academy of Sciences, 8 Arbuzov Str., 420088 Kazan, Russia; irek.rash@gmail.com (I.R.N.); kholin06@mail.ru (K.V.K.); sobaka-1968@mail.ru (A.D.V.); tatyanagr@gmail.com (T.P.G.); aidar@iopc.ru (A.T.G.); kamaka59@gmail.com (M.K.K.); rustem02@yandex.ru (R.R.Z.); asiyamust@mail.ru (A.R.M.)

[2] Department of Electronic Instrumentation and Quality Management, A.N. Tupolev Kazan Research Technological University, 420015 Kazan, Russia; iismaev@mail.ru

[3] Nikolaev Institute of Inorganic Chemistry, Siberian Branch of the Russian Academy of Sciences, 3 Acad. Lavrentiev Ave., 630090 Novosibirsk, Russia; kbrylev@gmail.com

* Correspondence: bulat_ahmadeev@mail.ru

Methods

Dynamic light scattering (DLS) measurements were performed by means of the Malvern Mastersize 2000 particle analyzer. A He–Ne laser operating at 633 nm wavelength and emitting vertically polarized lightwas used as a light source. The measured autocorrelation functions were analyzed by Malvern DTS software and the second-order cumulant expansion methods. The effective hydrodynamic radius (RH) was calculated by the Einstein–Stokes relation from the first cumulant: D = kBT/6πηRH, where D is the diffusion coefficient, kB is the Boltzmann constant, T is the absolute temperature, and η is the viscosity. The diffusion coefficient was measured at least three times for each sample. The average error in these experiments is approximately 4%.

Powder X-ray diffraction (PXRD) measurements were performed on a Bruker D8 Advance diffractometer equipped with a Vario attachment and Vantec linear PSD, using Cu radiation (40 kV, 40 mA) monochromated by a curved Johansson monochromator (λ Cu Kα1 1.5406 Å). Room-temperature data were collected in the reflection mode with a flat-plate sample. Sample was applied in liquid form on the surface of a standard zero diffraction silicon plate. After drying the layer, a few more layers were applied on top of it to increase the total amount of the sample. The sample was kept spinning (15 rpm) throughout the data collection. Patterns were recorded in the 2Θ range between 2° and 100°, in 0.008° steps, with a step time of 0.1–5.0 s. Several diffraction patterns in various experimental modes were collected for the sample. Processing of the data obtained was performed using EVA [1] and TOPAS [2]

software packages. The PDF-2 powder X-ray diffraction database (ICDD PDF-2, Release 2005–2009) was used to identify the crystalline phase.

Mn and Re were identified in the colloids using simultaneous inductively coupled plasma optical emission spectrometry (ICP-OES) model iCAP 6300 DUO by Varian Thermo Scientific Company equipped with a CID detector. This spectrometer enables the simultaneous measurement of peak heights within the 166 to 867 nm range. The optical resolution is b 0.007 nm to 200 nm. The working frequency is 27.12 MHz. Together, the radial and axial view configurations enable optimal peak height measurements with suppressed spectral noises. Concentration of Mn and Re ions was determined, respectively, by the spectral lines 257.610 nm and 221.426 nm.

TEM images were obtained by use of Hitachi HT7700 (Japan) at an accelerating voltage of 100 kV.

ESR

The ESR measurements were carried out on an X-band ELEXSYS E500 ESR spectrometer. Samples in quartz ampoules 1 mm in diameter were inserted into ER 4102ST cavity, after which the spectrometer was tuned and EPR spectra were recorded. Bruker E 035M teslameter was used to accurately g-factor determine.

IR

The infrared spectra were recorded on a Tensor 27 Fourier-transform spectrometer (Bruker) in a range of 4000–400 cm^{-1} with an optical resolution of 4 cm^{-1} and an accumulation of 32 scans using KBr pressed pellets.

X-ray powder diffraction (XRPD).

XRPD measurements were performed on an automatic Bruker D8 Advance diffractometer equipped with a Vario attachment and Vantec linear PSD using Cu radiation (40 kV, 40 mA) monochromated by a curved Johansson monochromator (λ CuKα1 1.5406 Å). Room-temperature data were collected in the reflection mode with a flat-plate sample. Samples were applied on the surface of a standard zero diffraction silicon plate, which reduces background scattering. Patterns were recorded in the 2θ range between 3° and 90° in 0.008° steps with a step time of 1s. The samples were spun (15 rpm) throughout the data collection. For the samples several diffractograms were obtained, which were summed. Processing of the obtained data performed using EVA [1] and TOPAS [2] software packages. The crystallite size calculations were performed during full powder pattern fitting procedure using the TOPAS software package in several ways: the values, calculated from the half-width of the reflections (LVol-FWHM) and the integrated reflection intensity (LVol-IB), are the volume-weighted values of the crystallite sizes, and the CrySizeL (CrySizeG) parameter is the size of the crystallites in the direction

perpendicular to the analyzed planes, with the Lorentz (Gauss) type of peak broadening. The data obtained are shown in Tables S1–S3, the results of the Rietveld refinement are shown in Figures S4–S6.

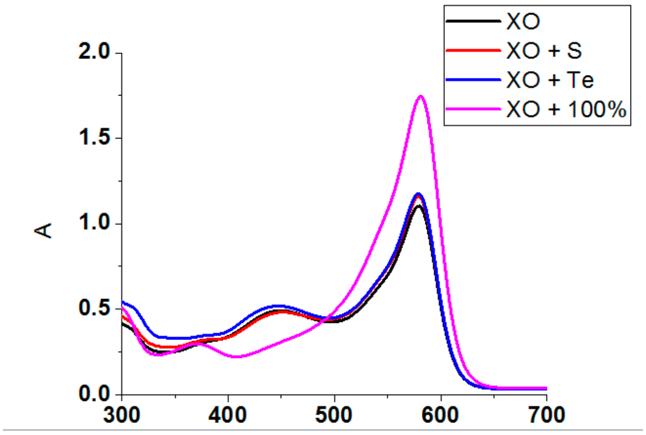

Figure S1. UV-vis spectra of Xylenole orange (black line), Xylenole orange with 1.17 mM Mn(II) (pink line), of Xylenole orange with supernatant, obtained after sedimentation $K_{4-2x}Mn_xRe_6Q_8$-based hydrophilic NPs: Q = S (red line), Q = Se^{2-} (blue line), Te(line).

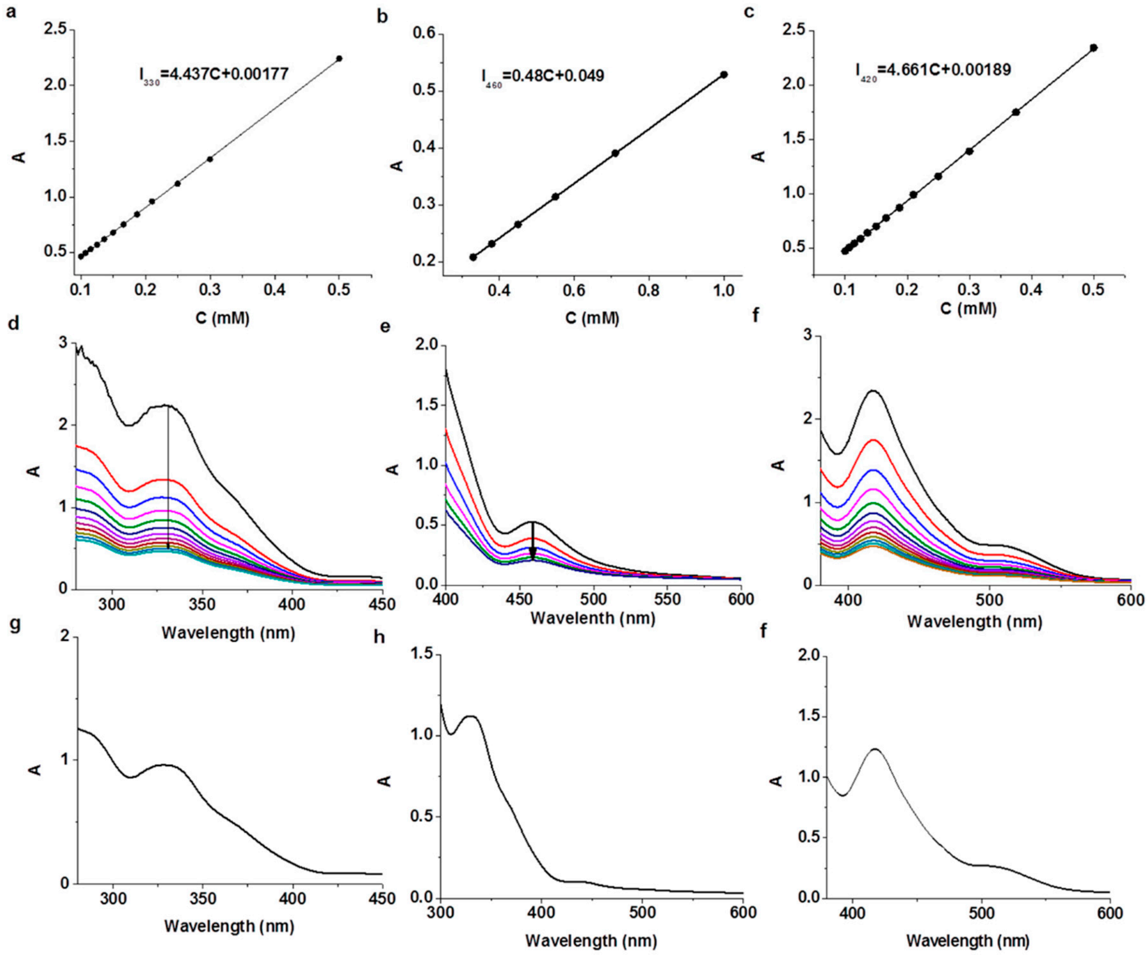

Figure S2. I/I_0 vs concentration of $[Re_6Q_8(CN)_6]^{4-}$ for Q = S (**a**), Se (**b**), Te (**c**). UV-vis spectra at different concentrations (concentration increase from purple to black line) of $Re_6Q_8(CN)_6]^{4-}$ for Q = S (**d**), Se (**e**), Te (**f**). UV-vis spetra of supernatant, obtained after centrifugation $K_{4-2x}Mn_xRe_6Q_8$-based hydrophilic NPs: Q = S^{2-} (**g**), Q = Se^{2-} (**h**), Te^{2-} (**f**).

Table S1. XO-assisted and ICP-OES analysis of $K_{4-2x}Mn_xRe_6Q_8$-based hydrophilic NPs

	Mn(II), mM	[Re6Q8(CN)6], mM	Ratio Mn:Re6
ICP-OES analysis	0.54	0.39 (Q = S)	1.38
	0.57	0.32 (Q = Se)	1.76
	0.55	0.31 (Q = Te)	1.79
XO-assisted analysis of supernatants	0.513	0.35(Q = S)	1.25
	0.49	0.31(Q = Se)	1.58

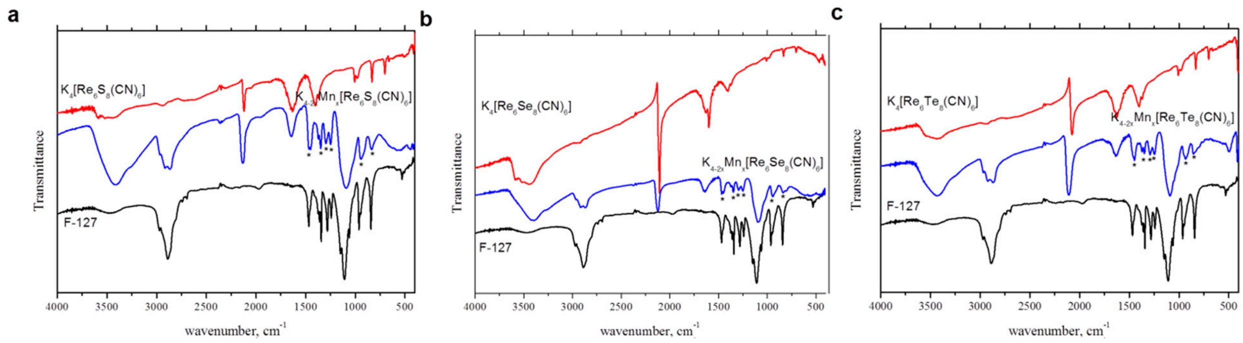

Figure S3. IR spectra of $K_{4-2x}Mn_xRe_6Q_8$-based NPs and $[Re_6Q_8(CN)_6]^{4-}$: Q = S^{2-} (**a**), Q = Se^{2-} (**b**), Te^{2-} (**c**).

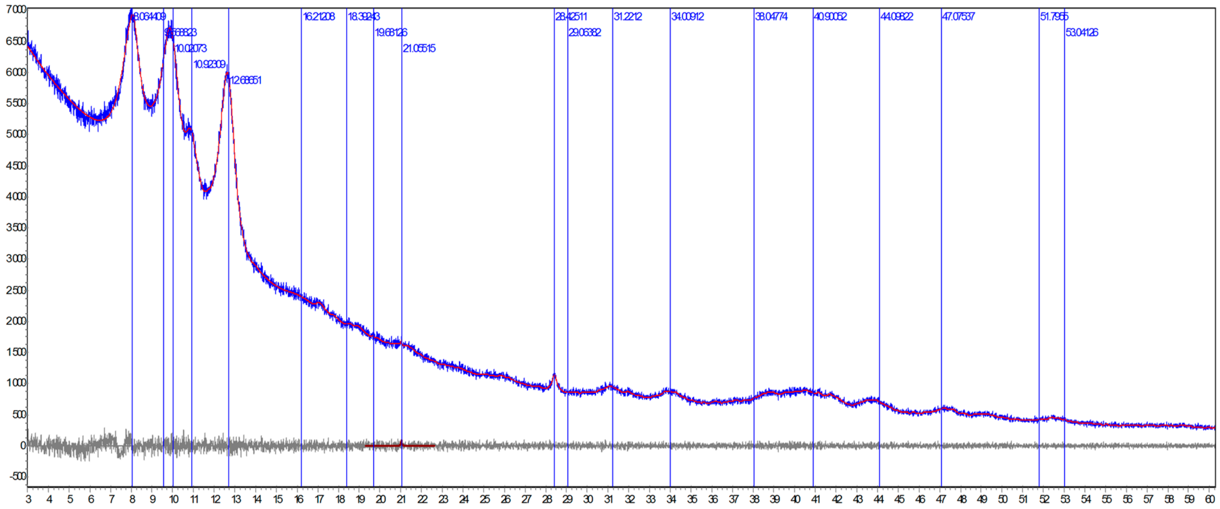

Figure S4. Powder XRD pattern of $K_{4-2x}Mn_xRe_6Q_8$, Q = Te^{2-} complex, experimental (blue), calculated (red) and their difference (grey) curves are depicted.

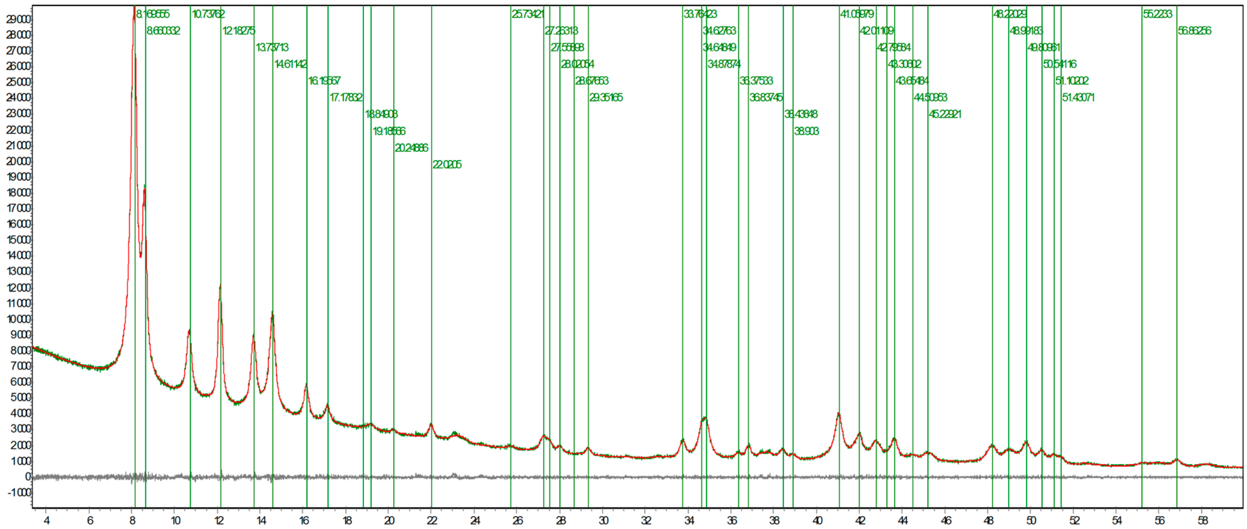

Figure S5. Powder XRD pattern of $K_{4-2x}Mn_xRe_6Q_8$, Q = Se^{2-} complex, experimental (green), calculated (red) and their difference (grey) curves are depicted.

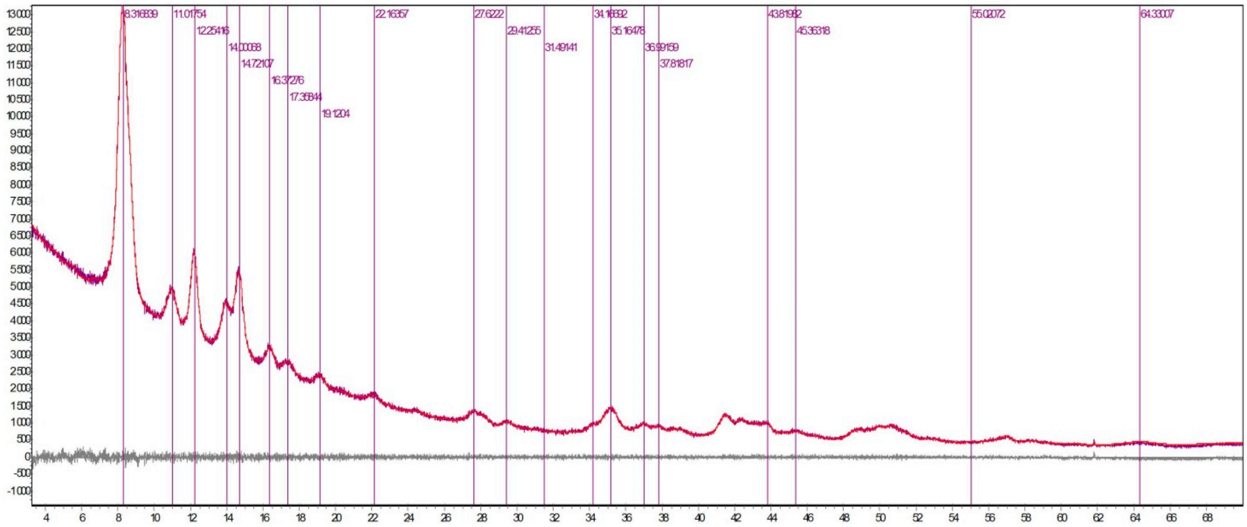

Figure S6. Powder XRD pattern of $K_{4-2x}Mn_xRe_6Q_8$, Q = S^{2-} complex, experimental (black), calculated (red) and their difference (grey) curves are depicted.

Table S2. Crystallite size, calculated from experimental diffraction patterns of $K_{4-2x}Mn_xRe_6Q_8$, Q = Se^{2-}.

**Angle 2θ, º**	8.1695(3)	8.6603(5)	10.737(1)	12.1827(5)	13.7371(9)	14.6114(8)
**I (a.u.)**	29.2(8)	10.7(1)	9.0(1)	15.9(2)	15.7(4)	28.1(4)
**CrySizeL (nm)**	32.8(2)	34.9(6)	35.5(2)	41.8(7)	34.9(7)	31.4(6)
**LVol-IB (nм)**	20.9(7)	31.3(9)	22.1(8)	29.7(9)	22.3(7)	18.6(7)
**Lvol-FWHM (nmм)**	29.2(5)	41.4(5)	30.5(4)	39.4(4)	31.1(4)	25.2(5)
**R_{wp}**	2.12%.					
**R_{exp}**	1.76%					

Table S3. Crystallite size, calculated from experimental diffraction patterns of $K_{4-2x}Mn_xRe_6Q_8$, Q = S^{2-} complex

**Angle 2θ, º**	8.311(3)	11.009(9)	12.240(1)	13.987(9)	14.708(6)	17.41(4)
**I(a.u.)**	21.6(3)	4.3(34)	9.6(3)	8.2(8)	16.2(6)	4.1(7)
**LVol-IB (nm)**	10.9(3)	11.6(6)	15.3(5)	12.0(6)	12.7(4)	11.0(5)
**Lvol-FWHM (nm)**	14.5(4)	13.0(5)	18.0(4)	12.7(5)	14.8(5)	11.5(4)
**R_{wp}**	2.74%.					
**R_{exp}**	2.54%					

Table S4. Crystallite size, calculated from experimental diffraction patterns of $K_{4-2x}Mn_xRe_6Q_8$, Q = Te^{2-}.

**Angle 2θ, º**	8.0915(5)	9.600(8)	10.002(5)	12.644(7)
**I (a.u.)**	12.50(8)	5.24(9)	4.50(3)	16.15(4)
**CrySizeL (nm)**	7.5(4)	10.5(5)	12.0(6)	12.2(5)
**LVol-IB (nm)**	6.7(9)	6.7(6)	11.8(5)	8.2(9)
**Lvol-FWHM (nm)**	6.7(2)	7.3(8)	11.3(8)	9.1(7)
**R_{wp}**	2.51%.			
**R_{exp}**	2.24%			

References

1. *DIFFRAC Plus Evaluation Package EVA, Version 11, User's Manual*; Bruker AXS: Karlsruhe, Germany, 2005; 258p.

2. *TOPAS V3: General Profile and Structure Analysis Software for Powder Diffraction Data*; Technical Reference; Bruker AXS: Karlsruhe. Germany, 2005; 117p.

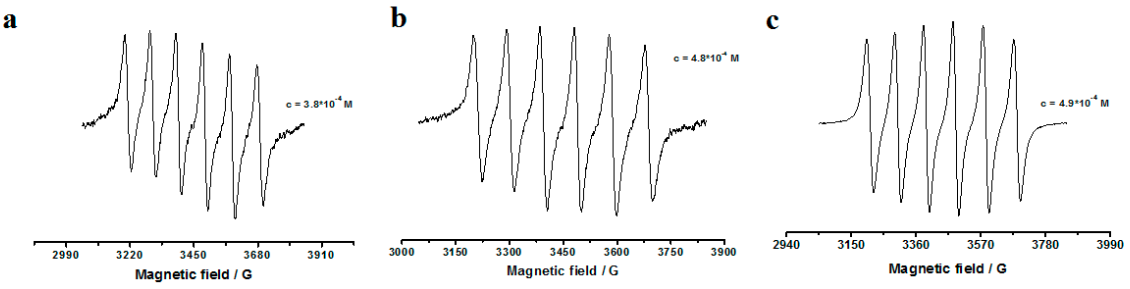

Figure S7. ESR spectra of of F-127-$K_{4-2x}Mn_xRe_6Q_8$: Q = S^{2-} (**a**); Q = Se^{2-} (**b**); Q = Te^{2-} (**c**).

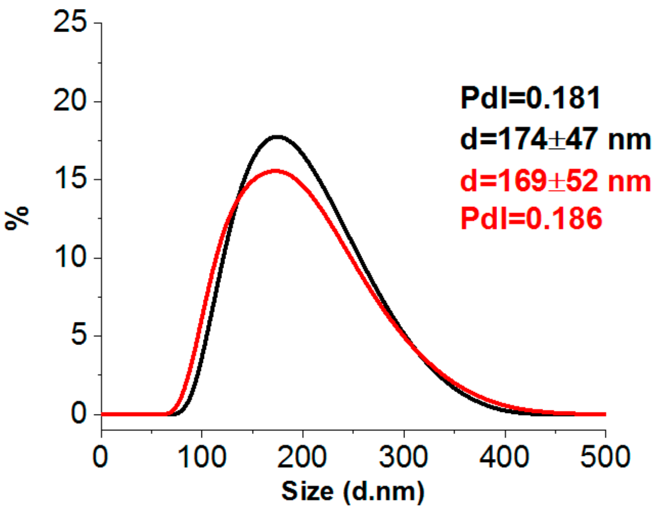

Figure S8. Size distribution by volume of F-127-$K_{4-2x}Mn_xRe_6Q_8$ at initial time (black line) and after 7 day (red line).

Table S5. Cell viability of F-127-$K_{4-2x}Mn_xRe_6Q_8$ at different manganese concentrations.

Compound	Concentration μM	Cell Viability%	M-HeLa IC_{50} μM
F-127– $K_{4-2x}Mn_xRe_6Se_8$	86.5	68.1	>86.5
	43.3	77.5	
	21.6	92.7	
	10.8	100	
	5.45	100	
	2.7	100	
F-127– $K_{4-2x}Mn_xRe_6S_8$	90.5	53.7	>90.5
	45.3	77.0	
	22.6	80.4	
	11.3	82.2	
	5.7	84.8	
	2.8	88.0	
**Compound**	**Concentration μM**	**Cell Viability%**	**Chang liver IC_{50} μM**
F-127– $K_{4-2x}Mn_xRe_6Se_8$	86.5	62.6	>90.0
	43.3	65	
	21.6	86.8	
	10.8	100	

	5.45	100	
	2.7	100	
F-127– $K_{4-2x}Mn_xRe_6S_8$	90.5	39.1	>91.5
	45.3	62.1	
	22.6	72.6	
	11.3	74.4	
	5.7	100	
	2.8	100	
